# Reply: UFT/leucovorin and oxaliplatin alternated with UFT/leucovorin and irinotecan in metastatic colorectal cancer

**DOI:** 10.1038/sj.bjc.6602017

**Published:** 2004-07-06

**Authors:** G Francini, R Petrioli

**Affiliations:** 1 Department of Human Pathology and Oncology; Medical Oncology section, University of Siena, Viale Bracci 11, 53100 Siena, Italy

**Sir,**

We would like to thank Dr Alliot for his comments concerning our study of metastatic colorectal cancer patients treated with UFT/LV+L-HOP alternated with UFT/LV+CPT-11 (Petrioli *et al*, 2004).

In relation to the baseline characteristics of the enrolled patients, it was pointed out in the Discussion that ‘the performance status and the percentage of chemonaive patients suggested a better than average group with regard to efficacy and toxicity’. As far as metastatic sites are concerned, a selection bias is common to small phase II studies. It is also worth pointing out that most of the metastatic colorectal cancer patients enrolled in clinical trials have ⩽2 metastatic sites (as in our study population). Furthermore, patients undergoing surgery for colorectal cancer are unlikely to have metastatic sites other than the liver, lung and peritoneum, and those having three or more metastatic sites are unlikely to have a performance status that would allow their enrolment in a chemotherapeutic protocol. Finally, phase II studies do not usually report baseline CEA, albumin and LDH levels because these have little prognostic value in the case of patients with advanced disease (Douillard *et al*, 2000; Saltz *et al*, 2000; Souglakos *et al*, 2002; Zeuli *et al*, 2003).

In relation to the low level of toxicity, it should be remembered that this was also due to the advantage of oral chemotherapy: that is, unlike boluses and continuous infusions, the treatment can be discontinued when toxicity arises and before it worsens (Twelves and Cassidy, 2002).

About 20% of our patients underwent postchemotherapy surgery for residual metastases, thus confirming the efficacy of the proposed chemotherapy protocol. However, postchemotherapy surgery led to a major advantage in terms of global survival in very few cases (9%).

The fact that 85% of our patients underwent primary tumour surgery is said to be a major recruitment bias, but we would like to point out that this percentage is the same or lower than that reported in the majority of studies of metastatic colorectal cancer (Van Cutsem *et al*, 2001; Twelves *et al*, 2001; Schilsky *et al*, 2002; Falcone *et al*, 2002).

As mentioned in the Discussion, low dose intensities of L-HOP and CPT-11 can be expected when using an alternating chemotherapy regimen. Nevertheless, the results of the study are supported by other studies of alternating chemotherapy in patients with metastatic colorectal cancer, suggesting that prolonged tumour exposure to a fluoropyrimidine plus full doses of L-HOP alternated with full doses of CPT-11 can be effective and well tolerated (Van Cutsem *et al*, 1998; Reina *et al*, 2003; Aparicio *et al*, 2003). Furthermore, it needs to be remembered that an aggressive chemotherapeutic approach can lead to numerous dose adjustments and delays, which inevitably lower the planned dose intensities, and it is difficult to continue the treatment over time (Falcone *et al*, 2002; Souglakos *et al*, 2002; Ychou *et al*, 2003).

Concerning the possibility of using higher doses of UFT, we would like to point out that we chose a fixed dose of 250 mg m^−2^ day^−1^ for 28 days on the basis of the activity and tolerability of UFT/LV+L-HOP or UFT/LV+CPT-11 demonstrated by previous phase I/II studies (Price and Hill, 2000; Vanhoefer and Wilke, 2001). The risk of overlapping diarrhoea is certainly greater with the combination of UFT/LV and CPT-11, and so higher UFT doses could perhaps be used in combination with L-HOP. However, other studies are difficult to interpret in this sense because they used shorter UFT administration schedules (21 days), which reduce the risk of diarrhoea (Kim *et al*, 2002; MacKay *et al*, 2003).

We believe that the hypothesis that more patients might have benefited from heavier regimens is difficult to support. Although 54% of the patients were candidates for a second surgical intervention, the albeit low 7.3% rate of complete remission (CR), which is considered the main indicator of treatment efficacy, was similar to that observed with much more aggressive chemotherapy regimens in patients with similar characteristics to those of our study population (Buyse *et al*, 2000; Saltz *et al*, 2000; Falcone *et al*, 2002; Souglakos *et al*, 2002). This suggests that the CR rate is more related to the biological characteristics of the tumour than the chemotherapy dose. We have obtained similar CR rates in previous clinical trials using the simple schedule of folinic acid and 5-fluorouracil (Francini *et al*, 1988; Petrioli *et al*, 1995).

In relation to the possible selection of resistant clones, the rationale of an alternating schedule is to ensure that full doses of all active agents can be administered by avoiding the potential incidence of overlapping side effects that may occur with concomitant drug administration. Our results suggest that a treatment based on low dose intensities of the more active drugs can be administered for a long time with a high level of tolerability, and similar response, time to progression and survival rates to those observed using more aggressive chemotherapy regimens (Douillard *et al*, 2000; Saltz *et al*, 2000; Souglakos *et al*, 2002).

Dr Alliot also raises the question of second-line chemotherapy, but this is a known problem when more active drugs are used. Furthermore, the results of second-line chemotherapies are generally unsatisfactory, whereas the simultaneous use of all of the drugs active in first-line treatment is an attempt to improve prognosis and many studies are investigating this approach (Falcone *et al*, 2002; Ychou *et al*, 2003).

Finally, we agree that, in a subgroup of patients with initially unresectable metastatic colorectal cancer, active first-line chemotherapy may permit secondary radical surgery of metastases, and this was also possible in our study population (Bismuth *et al*, 1996). Furthermore, neoadjuvant treatments play an important role in all types of neoplasms. However, the aim of our study was not to develop a neoadjuvant treatment but a new chemotherapy regimen that could improve patient compliance and reduce side effects. In the absence of phase I data concerning the recommended dose of a three-drug combination, we chose to alternate the previously explored combinations of UFT/LV+CPT-11 and UFT/LV+L-HOP and, albeit with all of the limitations of a small phase II study, the results seemed to be interesting because of the association of efficacy and little toxicity.
